# Kaposi Sarcoma Involving Kidney Allografts: A Report of Two Cases From Qatar and Literature Review

**DOI:** 10.7759/cureus.71573

**Published:** 2024-10-15

**Authors:** Mostafa Elshirbeny, Khaled Murshed, Ashraf Fawzy, Awais Nauman, Ahmed Hamdi, Mohammed Akhtar, Hassan Al-Malki, Mohamad Alkadi

**Affiliations:** 1 Nephrology, Hamad Medical Corporation, Doha, QAT; 2 Laboratory Medicine and Pathology, Hamad Medical Corporation, Doha, QAT

**Keywords:** end-stage kidney disease, kaposi sarcoma, kidney transplantation, post-transplant malignancy, renal involvement

## Abstract

Immunosuppression in kidney transplantation elevates the risk of malignancies, particularly immune-driven and virus-related cancers like Kaposi sarcoma (KS). KS typically manifests as single or multiple skin lesions following kidney transplantation but can also affect other organs. Involvement of the kidney allograft by KS is exceptionally rare, with only a few cases documented. In this report, we present all known cases of KS involving kidney allografts in adult transplant recipients in Qatar, accompanied by a brief review of the literature.

## Introduction

Immunosuppressive therapy in kidney transplant recipients significantly reduces the risk of allograft rejection but increases the risk of malignancy by approximately 20-fold compared to the general population [1]. The types of malignancies vary by geographic region; in Western countries such as North America, Europe, Australia, and New Zealand, skin cancers - specifically squamous cell carcinoma, basal cell carcinoma, and melanoma - are the most common. In contrast, Kaposi sarcoma (KS) is more prevalent in Africa and the Mediterranean. Overall cancer risk in transplant recipients is driven by cancer type, with the highest risks seen in viral-related and immune-mediated malignancies, such as post-transplant lymphoproliferative disorder, anogenital cancers, and KS [2-5].

In kidney transplant recipients, KS typically presents as single or multiple skin lesions, but it can also affect mucosal surfaces, including the oral cavity, gastrointestinal tract, lungs, and lymphoid tissues [6-8]. Kidney allograft involvement in KS is exceedingly rare, with only nine cases reported in the literature, including one from Qatar [9-17]. This report aims to present all known cases of KS involving kidney allografts in adult transplant recipients in Qatar, review the global literature, and provide a brief overview of the pathogenesis of KS in transplant recipients.

## Case presentation

Case 1

A 49-year-old male from Sudan with a history of end-stage kidney disease (ESKD) secondary to hypertension underwent a living unrelated kidney transplant in October 2016 in Egypt, with an uneventful postoperative course. No data were available regarding the donor or induction therapy. The patient was maintained on tacrolimus, mycophenolate mofetil (MMF), and prednisolone. He began follow-up at Hamad General Hospital transplant clinics in December 2016. His baseline serum creatinine level post-transplant was 95 µmol/L.

Five months after the kidney transplant, the patient’s kidney function began to deteriorate, with his serum creatinine rising to 152 µmol/L. His tacrolimus levels were persistently high post-transplant, ranging between 11 ng/mL and 18 ng/mL, despite several dosage adjustments. The patient remained asymptomatic, and his clinical examination and systemic review were unremarkable. There was no significant proteinuria or hematuria. Immune profile and virology screening revealed cytomegalovirus (CMV) viremia (CMV PCR: 1,327 IU/mL), while BK PCR was negative.

The patient underwent a kidney biopsy, which revealed moderate tubular toxic changes without evidence of T-cell-mediated or antibody-mediated rejection (C4d negative). Three months later, he presented with diarrhea, shortness of breath, and decreased urine output and was found to have acute allograft dysfunction, with a serum creatinine level of 700 µmol/L, hematuria, and proteinuria (urine protein to creatinine ratio of 52 mg/mmol). His tacrolimus level had decreased to 5 ng/mL. CMV PCR levels were elevated again at 5,138 IU/mL, while BK and Epstein-Barr virus (EBV) PCRs were negative.

**Figure 1:**
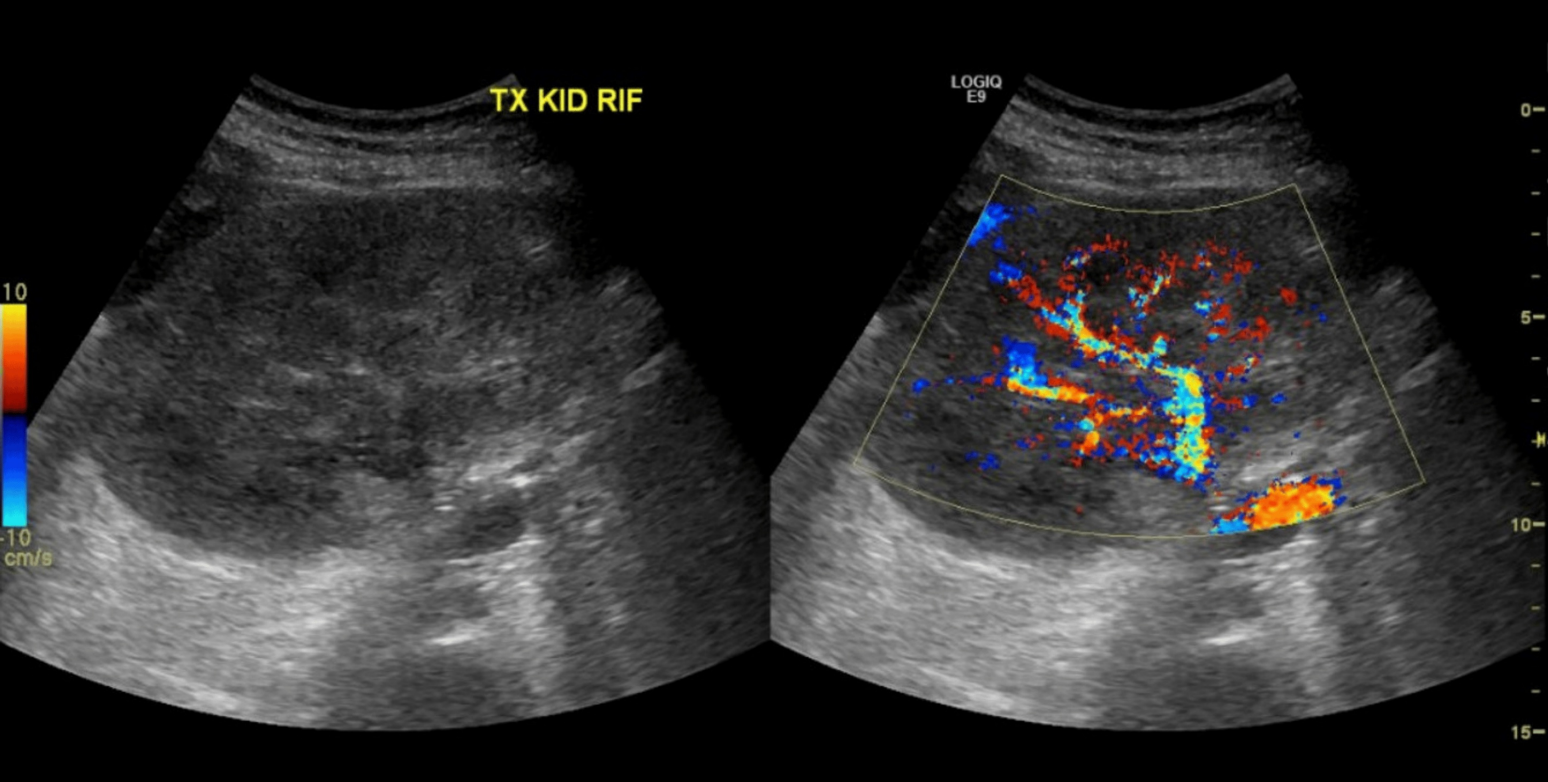
Case 1: Abdominal ultrasound The transplanted kidney, located in the right iliac fossa, is enlarged, measuring 13.7 × 6.3 × 7.2 cm, with an approximate volume of 326 cc.

Abdominal ultrasonography revealed an echogenic, diffusely enlarged transplanted kidney (Figure 1), with a size of 13.7 cm compared to 11.8 cm three months earlier. Hemodialysis (HD) was initiated due to volume overload and refractory metabolic acidosis. The patient was also started on IV ganciclovir for suspected CMV colitis.

**Figure 2:**
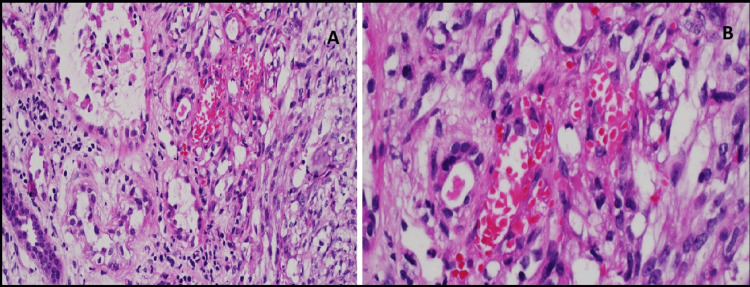
Case 1: Biopsy results (A) Photomicrograph showing an atypical spindle cell tumor infiltrating the renal parenchyma. (B) Tumor cells are spindle-shaped and form slit-like spaces containing extravasated red blood cells.

**Figure 3:**
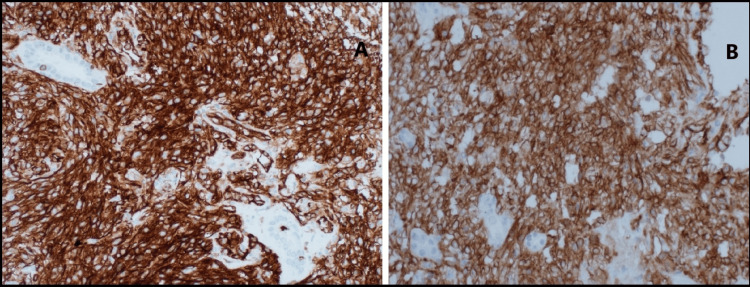
Case 1: Biopsy results (A) The tumor cells demonstrate immunoreactivity for CD34. (B) Additionally, they are positive for D2-40.

**Figure 4:**
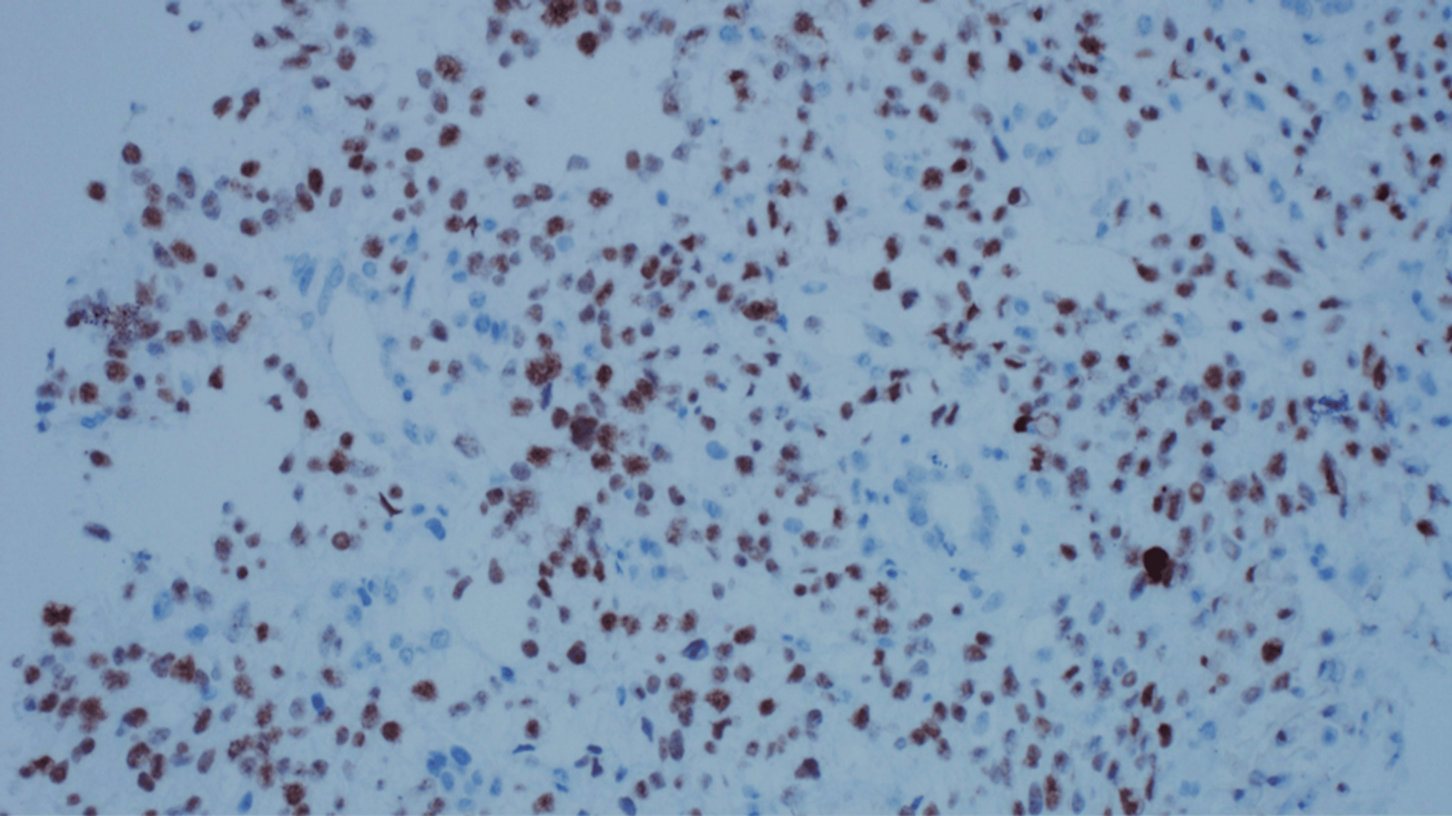
Case 1: Biopsy results The image demonstrates nuclear staining for HHV8. HHV8, human herpesvirus 8

A repeat kidney biopsy revealed an atypical spindle cell tumor forming slit-like spaces with extravasated red blood cells, extensively infiltrating the renal parenchyma (Figure 2A, 2B). The tumor cells were diffusely and strongly positive for CD34 and D2-40 (Figure 3A, 3B), and anti-human herpesvirus 8 (HHV8) staining showed positive granular nuclear staining (Figure 4).

**Figure 5:**
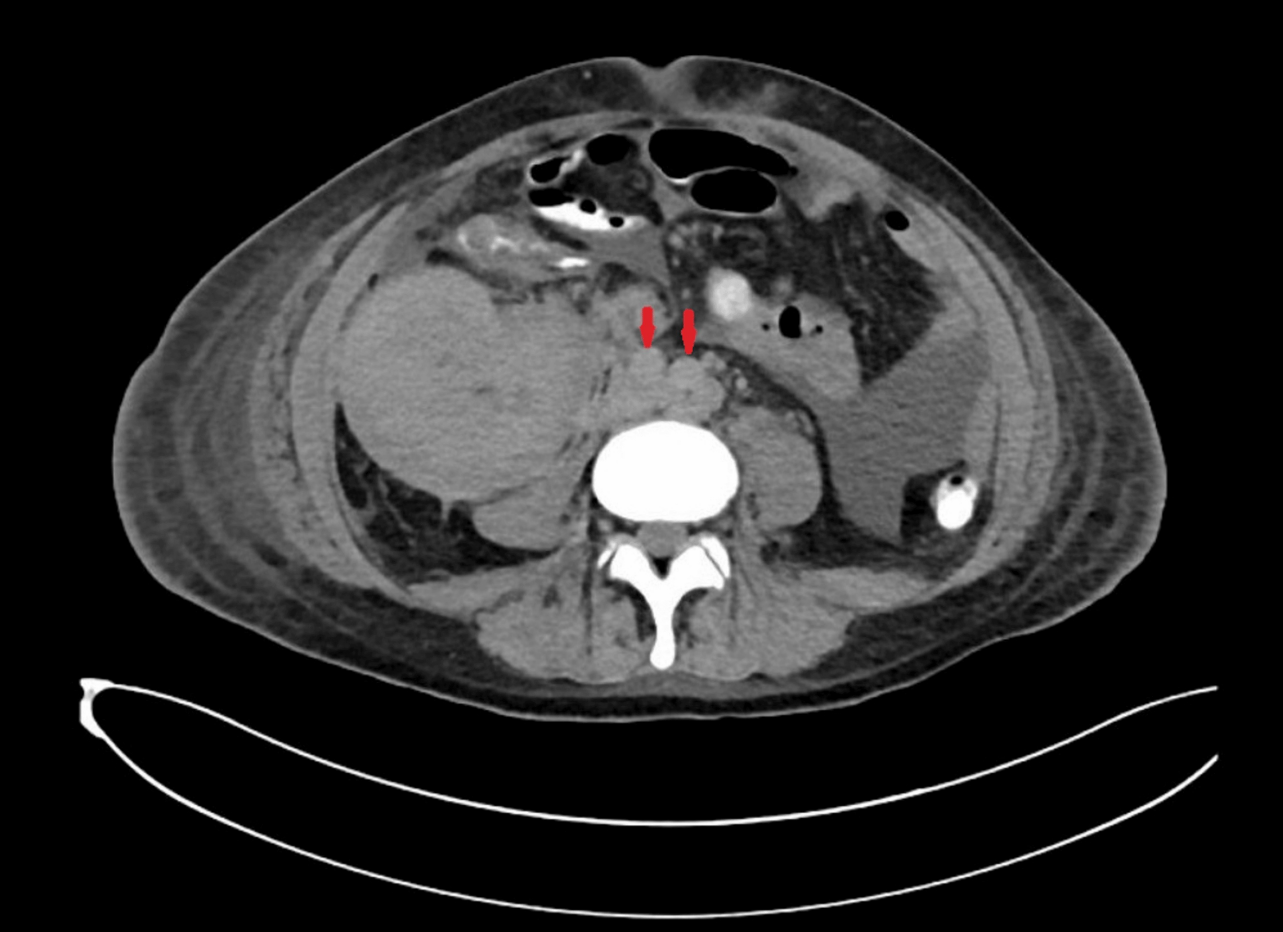
Case 1: CT abdomen The imaging reveals a bulky, non-functioning transplanted kidney exhibiting abnormal enhancement and multiple lymphadenopathies.

A CT scan with contrast was performed and revealed an enlarged transplanted kidney along with multiple enlarged retroperitoneal lymph nodes (Figure 5). The patient was diagnosed with KS involving the kidney allograft. His tacrolimus and MMF were discontinued, and he began treatment with liposomal doxorubicin administered every two weeks. Two weeks later, the patient began to pass urine and was switched to sirolimus 2 mg daily. His serum creatinine levels gradually improved, and HD was discontinued after four weeks of treatment.

**Figure 6:**
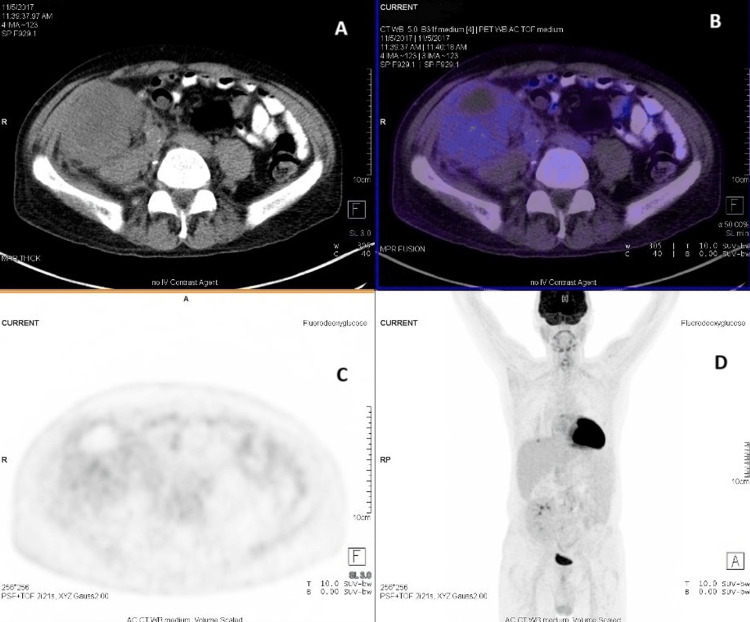
Case 1: PET-CT (A) The transplanted kidney is located in the right iliac fossa, showing decreased swelling (AP diameter reduced from 9 cm to 7 cm). (B) Evidence of FDG excretion indicates renal function. (C) Resolution of perinephric pathological soft tissue and enlarged paraaortic/right parailiac lymph nodes is observed. (D) No macroscopic signs of malignancy are suspected. FDG, fluorodeoxyglucose

The patient was discharged with a serum creatinine of 162 µmol/L and a sirolimus level of 4 ng/mL. He continued receiving doxorubicin every two weeks as an outpatient and completed a total of nine cycles. A PET-CT scan conducted five months post-discharge showed a reduction in the size of the transplanted kidney and resolution of perinephric pathological soft tissue, as well as a decrease in the size of the paraaortic and right parailiac lymph nodes (Figure 6). The frequency of doxorubicin administration was then reduced to every four weeks. The patient’s last serum creatinine, measured four years after discontinuing dialysis, was 123 µmol/L.

Case 2

A 46-year-old gentleman from Mozambique with a history of ESKD secondary to membranous nephropathy underwent a living, unrelated kidney transplant from his wife in April 2022 in Qatar, with an uneventful postoperative course. The patient received anti-thymocyte globulin induction therapy and was maintained on tacrolimus, MMF, and prednisolone. His baseline serum creatinine was 100 µmol/L. Two months post-transplant, his kidney function began to decline, with serum creatinine rising to 168 µmol/L. A kidney biopsy showed no significant findings, with no evidence of acute cellular or antibody-mediated rejection (C4d negative).

Six months later, the patient presented with decreased urine output and abdominal distension for three days, and acute graft dysfunction was noted, with a serum creatinine level of 241 µmol/L. CMV PCR was elevated at 948 IU/mL, while BK and EBV PCRs were negative. The patient was started on oral valganciclovir, and MMF was discontinued. Cell-free donor-derived DNA was high at 2.2%, prompting the administration of IV methylprednisolone 500 mg daily for three days. He was subsequently maintained on oral prednisolone 60 mg daily. By the time of discharge, his serum creatinine had decreased to 137 µmol/L.

**Figure 7:**
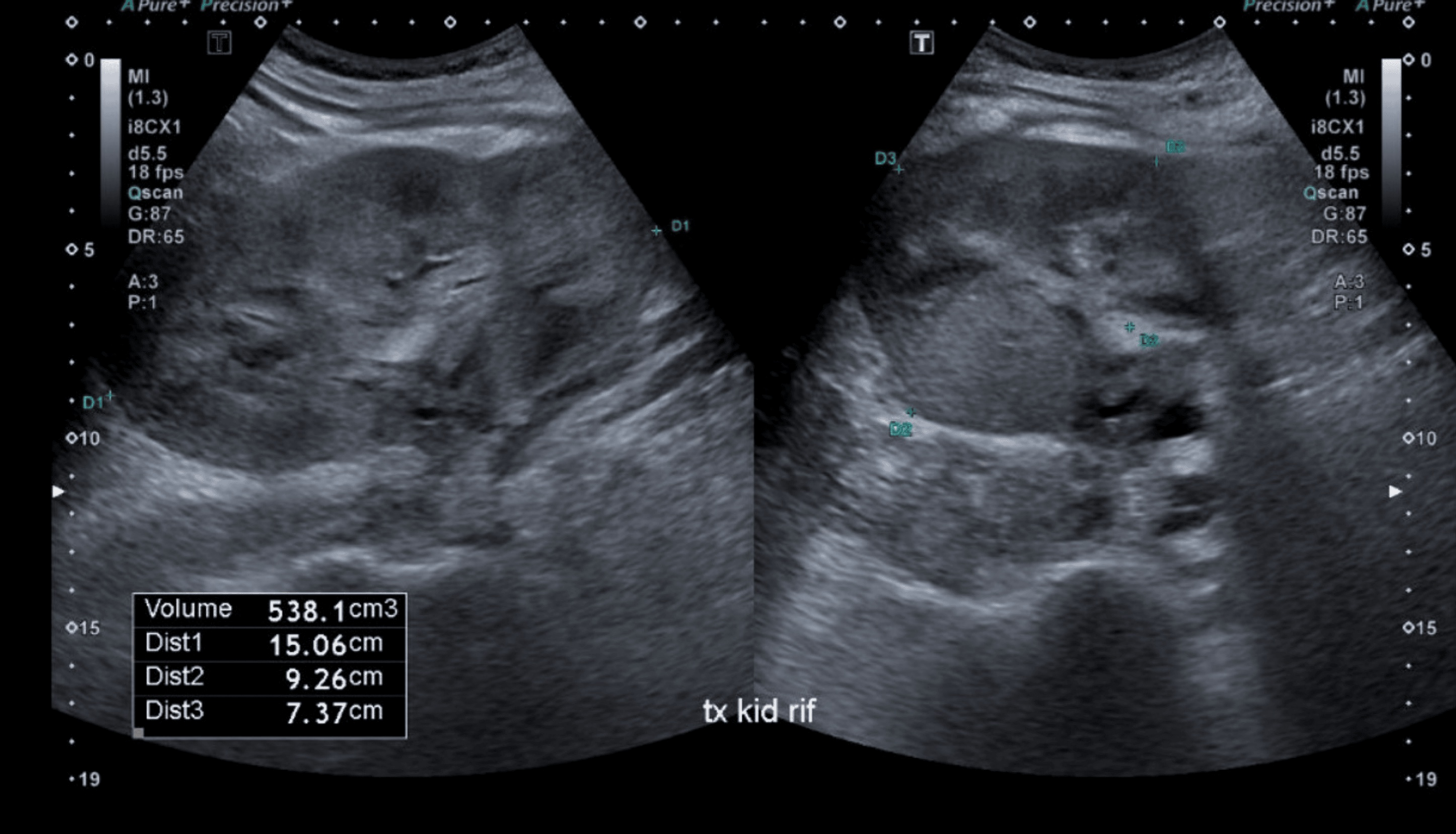
Case 2: Abdominal ultrasound The imaging shows an enlarged transplanted kidney with an approximate volume of 538 cc.

The patient then traveled to Mozambique for two months, and shortly after returning to Qatar, he was admitted for acute graft dysfunction with a serum creatinine level of 335 µmol/L and pancytopenia (WBC: 1.7 × 10^3^/uL, hemoglobin: 10.2 g/dL, hematocrit: 32.4%, and platelet count: 91 × 10^3^/uL). A urinary tract ultrasound revealed an echogenic, diffusely enlarged transplanted kidney (Figure 7; kidney size increased to 15 cm from 12 cm three months prior). The initial diagnosis was possible thrombotic microangiopathy (TMA) secondary to tacrolimus. Tacrolimus was discontinued and replaced with sirolimus, and plasmapheresis sessions were started due to suspected TMA and worsening kidney function.

**Figure 8:**
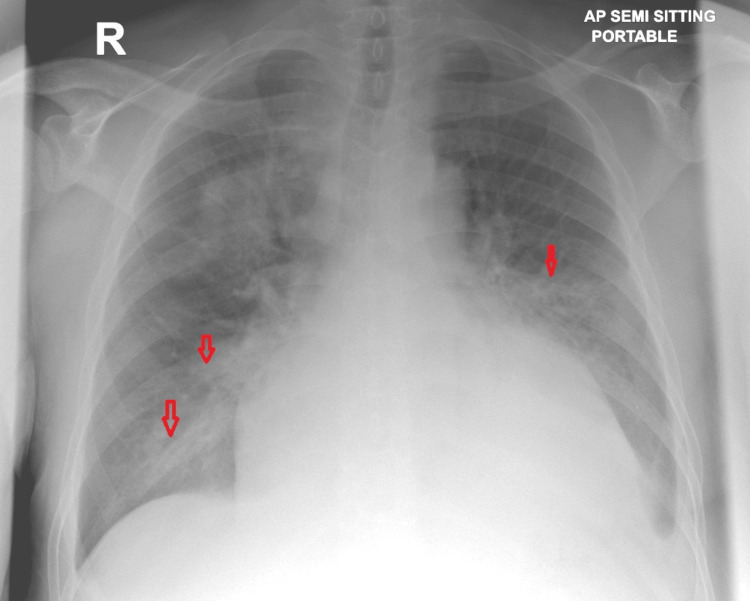
Case 2: Chest X-ray The chest X-ray reveals an enlarged heart, bilateral pleural effusions, and bilateral perihilar and mid-to-lower zone lung consolidation.

**Figure 9:**
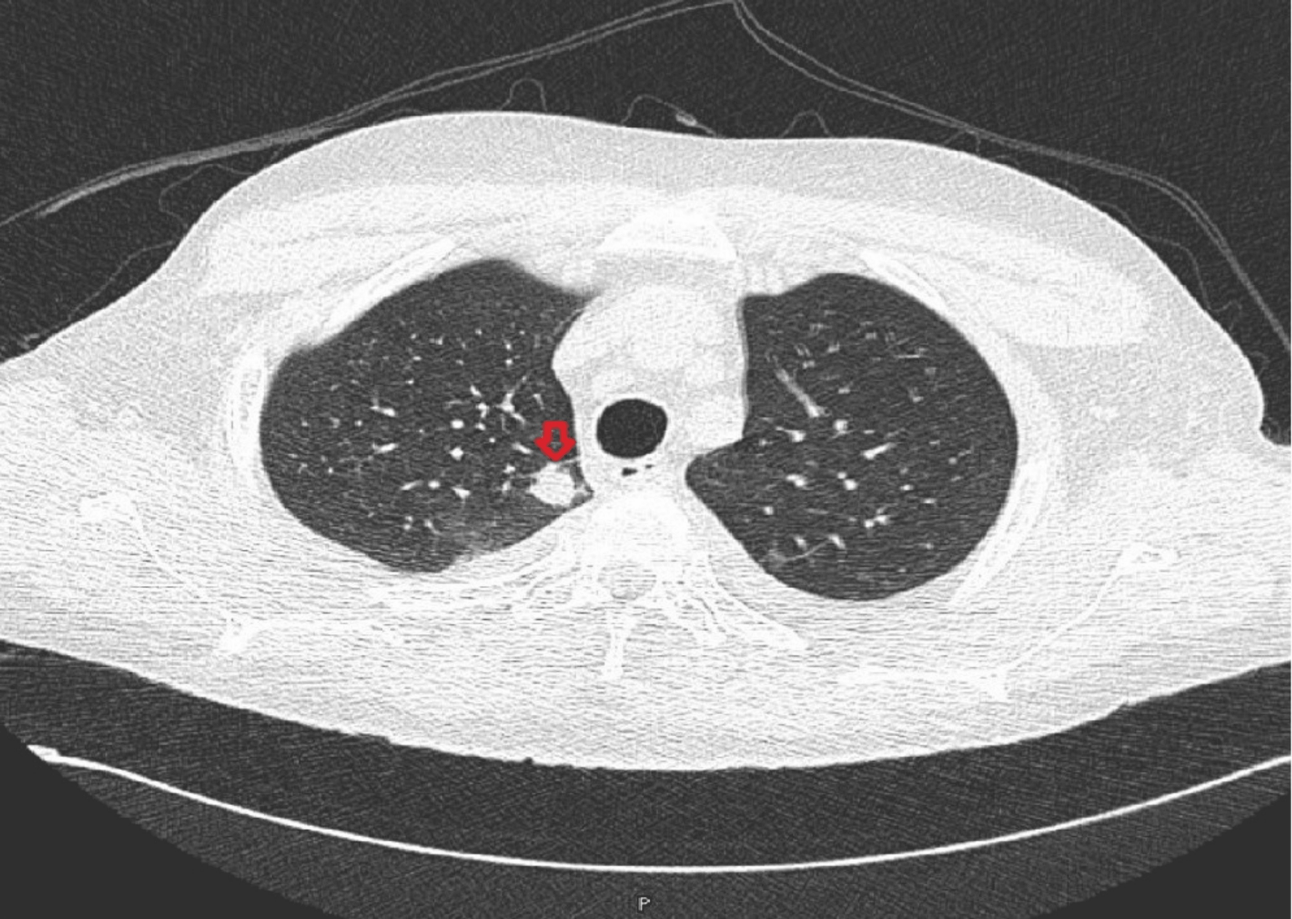
Case 2: Chest CT The chest CT scan reveals multiple low-density necrotizing pulmonary nodules in the right lung as well as a solitary cavitary nodule.

**Figure 10:**
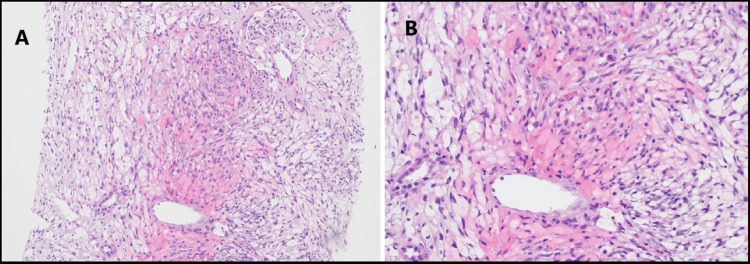
Case 2: Biopsy results (A) Photomicrograph depicting a spindle cell tumor infiltrating the renal interstitium while preserving the glomeruli. (B) The tumor cells are spindle-shaped, exhibit mild nuclear atypia, and form slit-like spaces.

**Figure 11:**
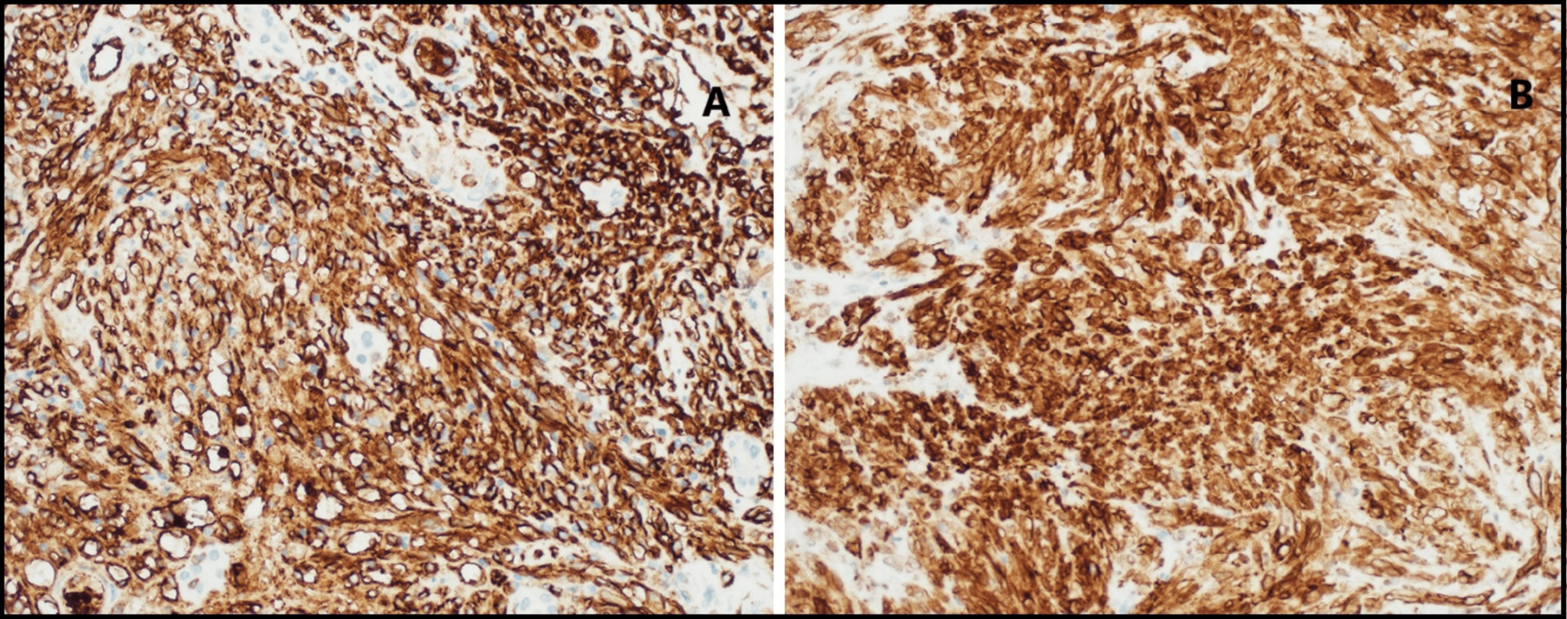
Case 2: Biopsy results (A) The tumor cells demonstrate immunoreactivity for CD34. (B) The tumor cells are also positive for D2-40.

**Figure 12:**
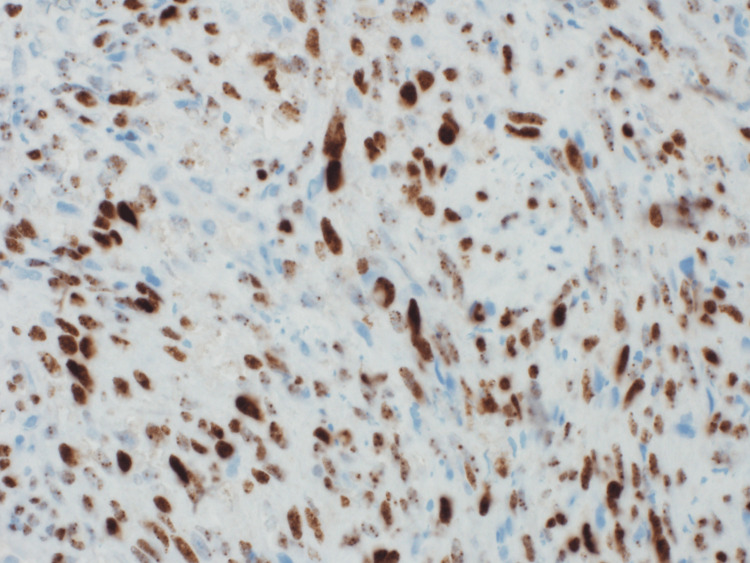
Case 2: Biopsy results The biopsy demonstrates nuclear staining for HHV8. HHV8, human herpesvirus 8

Subsequently, the patient developed chest pain, and a chest X-ray showed new bilateral patchy infiltrates (Figure 8). A CT scan of the chest revealed multiple bilateral pulmonary nodules (Figure 9). After the patient’s platelet count improved, a kidney allograft biopsy was performed, showing a tumor infiltrating the renal parenchyma, composed of mildly atypical spindle cells arranged in fascicles with slit-like spaces, extravasated red blood cells, and plasma cells. Focal areas of necrosis were also noted (Figure 10A, 10B). More than 80% of the biopsy tissue was involved in the tumor. Immunohistochemical staining revealed that the tumor cells were diffusely and strongly positive for CD34 and D2-40 (Figure 11A, 11B). HHV8 showed positive granular nuclear staining (Figure 12), while C4d and SV-40 stains were negative.

**Figure 13:**
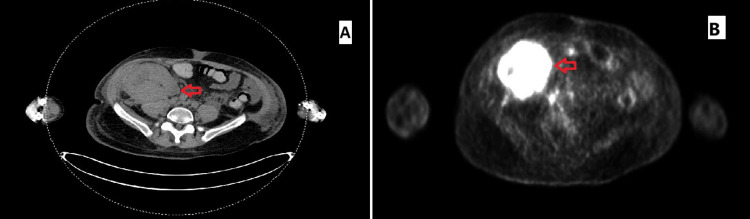
Case 2: PET-CT (A) The transplanted kidney is enlarged, indicating biopsy-proven KS. (B) The imaging shows intense, inhomogeneous uptake. KS, Kaposi sarcoma

A PET-CT scan was performed, revealing intense uptake in the enlarged transplanted kidney, along with lymph nodal, prostate, and left testicular involvements (Figure 13). The patient’s kidney function further deteriorated, necessitating the initiation of HD due to volume overload. Subsequently, the patient decided to return to his home country to complete his treatment while continuing on sirolimus.

## Discussion

KS was first described in 1872 by Moritz Kaposi, a Hungarian physician and dermatologist specializing in dermatopathology. While practicing in Vienna, Dr. Kaposi identified several cases of multifocal, pigmented vascular skin lesions in elderly European men. These lesions were aggressive, leading to death within two years [18]. This form of KS, now referred to as classic KS, predominantly affects elderly men of Jewish or Mediterranean descent. It mainly involves the skin of the legs and, contrary to the early descriptions, typically follows a slow, protracted course.

An endemic form of KS was later identified, primarily affecting African men aged 20-50, causing large nodules on the lower extremities. This form may progress rapidly, remain stable for years, or spontaneously resolve [19]. Another highly aggressive form emerged during the AIDS epidemic in the 1980s, known as AIDS-related or epidemic KS [19,20]. Additionally, KS can develop in individuals with immunosuppression due to medical treatment, such as organ transplant recipients, a form known as iatrogenic KS [21].

The incidence of KS in renal transplant recipients varies geographically. In some Middle Eastern countries, KS is the most common post-transplant malignancy [22-24]. The development of iatrogenic KS in transplant recipients is influenced by factors such as male gender, older age, and the prevalence of classic KS in the region of origin of both recipients and donors. KS is strongly associated with HHV8, also called KS herpesvirus (KSHV), with the highest prevalence seen in sub-Saharan Africa and the Amazon River delta, where infection rates exceed 90% in some adult populations. In the Mediterranean, prevalence ranges from 20% to 30%, while it is less than 10% in northern Europe, Asia, and the United States [25].

 Although our first patient was from Sudan, outside sub-Saharan Africa, the country still has a high incidence of KS, particularly post-transplant [26]. Our second patient was from Mozambique, a sub-Saharan country with a high endemic KS incidence.

In 1994, Chang et al. first identified KSHV in AIDS-related KS lesions using PCR amplification to compare affected and normal tissues [27]. KSHV is typically transmitted through saliva via close contact, as well as through blood, organ donation, and, rarely, from mother to fetus. The virus has both latent and lytic phases. During the latent phase, KSHV remains in host cells without causing pathology. However, under immunosuppressive conditions, the virus can switch to its lytic phase, during which viral replication and new virion assembly occur, potentially driving tumorigenesis by promoting cellular proliferation and neoplastic transformation [4,5].

KS is classified as a lymphatic/vascular neoplasm, although some debate exists about whether it represents a true sarcoma or a multifocal reactive hyperplasia of the vascular endothelium. In renal transplant recipients, KS typically presents as solitary or multiple skin lesions, with mucosal or visceral involvement in 25-50% of cases, often leading to a poor prognosis [16]. However, involvement of the renal allograft is exceedingly rare, with only nine cases reported in the literature (Table 1) [9-17]. Patients diagnosed with KS involving the kidney allograft range from 28 to 71 years of age, with most diagnoses occurring within the first year post-transplant. Tumor infiltration is commonly associated with graft dysfunction.

**Table 1:** Reported cases of KS involving the kidney allograft in kidney transplant recipients ADPKD, autosomal dominant polycystic kidney disease; Aza, azathioprine; CsA, cyclosporin A; DM, diabetes mellitus; GN, glomerulonephritis; HD, hemodialysis; HTN, hypertension; ICGN, immune-complex glomerulonephritis; IS, immunosuppressants; KS, Kaposi sarcoma; LN, lupus nephritis; MMF, mycophenolate mofetil; MN, membranous nephropathy; MPGN, membranoproliferative glomerulonephritis; mTOR, mammalian target of rapamycin; Pred, prednisolone; Tac, tacrolimus

Cases	Age	Sex	Native kidney disease	Donor type	IS regimen	Symptoms at presentation	Time to diagnosis	KS localization in renal allograft and urinary tract	Graft dysfunction	Treatment	Kidney and patient outcomes
Butkus et al. (1989) [9]	48	M	MPGN	Deceased	Pred, Aza, and CsA	Painless nodular lesion in left arm and chest	8 months	Renal parenchyma	No	Nephrectomy	Patient was returned to dialysis and all lesions had totally disappeared
Foulet et al. (1996) [10]	60	F	NA	Living	Pred, Aza, and CsA	Cutaneous lesions	14 months	Multiple nodules in renal parenchyma	Yes	IS withdrawal and nephrectomy	NA
Díaz-Candamio et al. (1998) [11]	51	M	ADPKD	Deceased	Pred, Aza, and CsA	Edema in the right lower leg and pain in the right groin	6 months	Mass at renal sinus and hilum	No	IS withdrawal with chemotherapy + surgery	Good renal function but the patient died nine months after diagnosis due to sepsis and malnutrition
Rha et al. (2000) [12]	28	F	LN	NA	Pred and CsA	Leg swelling, anorexia, and nausea	10 years	Multiple nodules in the ureter and renal pelvis	Yes	IS withdrawal and chemotherapy	Improving graft function; disappearance of all cutaneous and visceral lesions
Dudderidge et al. (2008) [13]	55	F	GN	Living	Pred, CsA, and MMF	Asymptomatic	12 months	Mass in the distal ureter	Yes	IS withdrawal	Improved kidney function and general condition
Markowitz et al. (2001) [14]	66	M	HTN	Deceased	Pred, CsA, and MMF	Asymptomatic	7 months	Renal parenchyma	Yes	IS withdrawal and chemotherapy	Improvement in kidney function after chemotherapy
Nauman et al. (2019) [15]	60	M	HTN	Living	Pred, Tac, and MMF	Vomiting	12 months	Renal parenchyma	Yes	mTOR	Maintained on HD; died after six years of diagnosis due to sepsis
Story et al. (2021) [16]	71	M	ICGN	Deceased	Pred, Tac, and MMF	Leg edema	5 months	Renal parenchyma	Yes	IS withdrawal and nephrectomy	Patient died 2.5 months after the diagnosis
Lee et al. (2023) [17]	53	F	DM	Deceased	Pred, Tac, and MMF	Asymptomatic	10 weeks	Renal parenchyma	Yes	Graftectomy	Maintained on HD; improved without chemotherapy
Our first case	49	M	DM	Living	Pred, Tac, and MMF	Diarrhea and decreased urine output	8 months	Renal parenchyma	Yes	IS withdrawal and chemotherapy	Recovery of kidney function; general improvement
Our second case	46	M	MN	Living	Pred, Tac, and MMF	Asymptomatic	10 months	Renal parenchyma	Yes	mTOR	Maintained on HD; lost to follow-up

The primary treatment for KS in renal transplant recipients involves reducing or discontinuing immunosuppression to curb viral replication [28], which can result in tumor regression and complete remission [29]. Another option involves replacing calcineurin inhibitors with sirolimus, an immunosuppressant that may also limit tumor progression [30]. Table 1 provides a summary of all reported cases of KS involving kidney allografts in transplant recipients, including our own cases.

## Conclusions

KS in kidney transplant recipients, although rare, remains a significant complication linked to the immunosuppressive therapy necessary to prevent graft rejection. This report from Qatar underscores the seriousness of KS involving the kidney allograft. The cases highlight the varied clinical presentations and the importance of individualized treatment strategies, such as reducing immunosuppression and considering specific chemotherapeutic options. Continued research is crucial to optimizing treatment protocols and enhancing patient outcomes.
